# JZTX-V Targets the Voltage Sensor in Kv4.2 to Inhibit I_to_ Potassium Channels in Cardiomyocytes

**DOI:** 10.3389/fphar.2019.00357

**Published:** 2019-04-16

**Authors:** Yiya Zhang, Ji Luo, Juan He, Mingqiang Rong, Xiongzhi Zeng

**Affiliations:** ^1^Department of Dermatology, Xiangya Hospital, Central South University, Changsha, China; ^2^Key Laboratory of Organ Injury, Aging and Regenerative Medicine of Hunan Province, Central South University, Changsha, China; ^3^The National and Local Joint Engineering Laboratory of Animal Peptide Drug Development, College of Life Sciences, Hunan Normal University, Changsha, China

**Keywords:** JXTX-V, cardiomyocytes, action potential, Kv4.2 channel, hydrophobic patch

## Abstract

Kv4 potassium channels are responsible for transient outward K^+^ currents in the cardiac action potential (AP). Previous experiments by our group demonstrated that Jingzhaotoxin-V (JZTX-V) selectively inhibits A-type potassium channels. However, the specific effects of JZTX-V on the transient outward (I_to_) current of cardiomyocytes and underlying mechanism of action remain unclear. In the current study, 100 nM JZTX-V effectively inhibited the I_to_ current and extended the action potential duration (APD) of neonatal rat ventricular myocytes (NRVM). We further analyzed the effects of JZTX-V on Kv4.2, a cloned channel believed to underlie the I_to_ current in rat cardiomyocytes. JZTX-V inhibited the Kv4.2 current with a half-maximal inhibitory concentration (IC_50_) of 13 ± 1.7 nM. To establish the molecular mechanism underlying the inhibitory action of JZTX-V on Kv4.2, we performed alanine scanning mutagenesis of Kv4.2 and JZTX-V and assessed the effects of the mutations on binding activities of the proteins. Interestingly, the Kv4.2 mutations V285A, F289A, and V290A reduced the affinity for JZTX-V while I275A and L277A increased the affinity for JZTX-V. Moreover, mutation of positively charged residues (R20 and K22) of JZTX-V and the hydrophobic patch (formed by W5, M6, and W7) led to a significant reduction in toxin sensitivity, indicating that the hydrophobic patch and electrostatic interactions played key roles in the binding of JZTX-V with Kv4.2. Data from our study have shed light on the specific roles and molecular mechanisms of JZTX-V in the regulation of I_to_ potassium channels and supported its utility as a potential novel antiarrhythmic drug.

## Introduction

Potassium channels play important roles in cellular signaling processes in both excitable and non-excitable cells. Voltage-gated K^+^ channels containing the central pore domain and voltage sensor domains (S1–S4 segments) regulate several fundamental physiological processes, including action potential (AP) repolarization, hormone secretion, and neurotransmitter release (Shieh et al., 2000). The voltage-gated Kv4 channel expressed in most mammalian cardiomyocytes plays a pivotal role in initial rapid repolarization (phase 1) and determines the amplitudes, and duration of cardiac AP by carrying the transient outward K^+^ current I_to_ (Stones et al., 2009). Moreover, I_to_ indirectly affects membrane repolarization by interfering with the subsequent ion currents, I_Ca_, I_Kr_, and I_Ks_, at phases 2 and 3 (Yue et al., 1997). Considering the critical involvement of I_to_ in action potential duration (APD), inhibitors of Kv4 channels have been reported to act as anti-arrhythmic agents via an ionic mechanism (Feng et al., 1997; Ma et al., 2015).

Animal toxins are important resources for the development of new therapeutic drugs due to their high selectivity and specificity for molecular targets (Zambelli et al., 2016). ω-conotoxin MVIIA (named Ziconotide) obtained from snail *Conus magus* was the first marine-derived drug produced in 2004 (Molinski et al., 2009). Eptifibatide extracted from venom of *Sistrurus miliarius barbourin* is effectively used for treatment of acute coronary syndromes (Tcheng and O’Shea, 1999; Zambelli et al., 2016). Exenatide, a peptide from *lizard Heloderma suspectum*, is currently used for treatment of type 2 diabetes (Goud et al., 2015). Spider venom contains peptide toxins targeting ion channels, making it a valuable resource for several potential therapeutic applications (Peng et al., 2017). For example, Lycosin-II and Lycosin-I, the peptide toxins from *Lycosa singoriensis* display high antibacterial activity against multidrug-resistant *Acinetobacter baumannii* (Wang et al., 2014, 2016). mu-TRTX-Hhn1b (HNTX-IV) from *Ornithoctonus hainana* and Huwentoxin-XVI from *O. huwena* exert analgesic effects against inflammatory and neuropathic pain by specifically inhibiting the voltage-gated Nav1.7 channel (Liu et al., 2014), and N-type calcium channel (Deng et al., 2014), respectively. Recent experiments have further demonstrated that Lycosin-I has vasodilator and hypotensive effects (Ma et al., 2018).

Jingzhaotoxin-V (JZTX-V), a 29-residue peptide isolated from venom of the Chinese tarantula *Chilobrachys jingzhao*, effectively inhibits the transient outward potassium channels in dorsal root ganglion neurons of adult rats while barely affecting the currents of outward delay rectifier potassium channels (Yuan et al., 2007; Zeng et al., 2007). However, the specific effects of this peptide on cardiomyocyte function remain to be established. In the current study, we have focused on the effects of JZTX-V on the I_to_ current and APD in neonatal rat ventricular myocytes (NRVM) and the mechanism of JZTX-V binding to the Kv4.2 channel. Our results collectively support the utility of JZTX-V as a potential novel antiarrhythmic drug.

## Materials and Methods

### JZTX-V Synthesis

Jingzhaotoxin-V and its mutants were synthesized using a solid-phase chemical approach with Fmoc-protected amino acids. The synthetic peptides were purified by reverse-phase HPLC and then subjected to oxidative refolding under the optimal conditions as previously described (Huang et al., 2014). The peptide toxins were used in laboratory only and the hazardous wastes were collected and sent for centralized treatment by Hunan Normal University.

### Ventricular Myocyte Isolation

Neonatal rat ventricular myocytes cells were obtained from ventricles of neonatal Sprague–Dawley rats as previously reported (Sung et al., 2012; Yan et al., 2018). SD rats (Hunan SJA Laboratory Animal Co., Ltd., Changsha, China) were used according to the guidelines of the National Institutes of Health for Care and Use of Laboratory Animals. The experiments were approved by the Animal Care and Use Committee of Hunan Normal University.

### Collection of the Venom

The venom from adult female *Jingzhao* spiders was collected by using an electro-pulse stimulator as previous described (Rong et al., 2011). The venom was used in laboratory only and the hazardous wastes were collected and sent for centralized treatment by Hunan Normal University.

### Circular Dichroism (CD)

30 μM synthetic JZTX-V and its mutants were used for the CD spectra from 260 to 190 nm using a Jasco J-725 spectropolarimeter (Jasco, Osaka, Japan) as previous describe (Rong et al., 2011).

### Expression of Kv4 Channels

Human embryonic kidney cell line (HEK293T) was obtained from Shanghai Institute of Cell Biology, Chinese Academy of Sciences (Shanghai, China) and maintained with 5% CO_2_ at 37°C in DMEM supplemented with 10% heat-inactivated fetal calf and serum (FBS), penicillin (100 U/ml), and streptomycin (100 μg/ml). The WT/mutant Kv4.2 plasmids were transiently transfected into HEK293T by Lipofectamine^TM^ 2000 Reagent (Invitrogen), and the KChIP2 subunits were co-transfected with Kv4.2. The Kv4.2 mutations were constructed using the GeneTailor^TM^ Site-Directed Mutagenesis System (Invitrogen, Carlsbad, CA, United States) as previously described (Rong et al., 2011).

### Homology Modeling

The DeepView program and Swiss-Model server were used for homology modeling. Three-dimensional models of ScTx1, JZTX-V, and Ctri8557 were obtained basing on ZTX-XI (PDB code 2a2v), PaTx1 (PDB code 1V7F), and Ia2 (PDB code 1lir), respectively.

### Patch Clamp Recording and Data Analysis

Whole-cell patch-clamp recordings were performed by an Axon 700B patch-clamp amplifier (Axon Instruments, Irvine, CA, United States) as previous described (Rong et al., 2011). The extracellular buffer and pipette solution for AP, I_to_, and Kv4.2 were used as follows.

The APs, Pipette solution contained (mM) 120 KCl, 1 MgCl_2_, 10 EGTA, 10 Hepes, and 3 MgATP (pH = 7.4). The extracellular buffer contained (mM) 140 NaCl, 5.4 KCl, 1.3 CaCl_2_, 0.5 MgCl_2_, 5 Hepes, and 5.5 glucose (pH = 7.4).

I_to_ currents, The Ca^2+^-currents blocker CdCl_2_ (200-μmol/L) were used in external solutions. To generate I_to1_ current, cells were held at −40 mV to inactivate the fast Na^+^ current and then stepped to potentials of −40 mV to +60 mV in 10-mV increments. For the inhibition of JZTX-V on I_to1_, I_to1_ current were elicited by +30 mV depolarizing voltage from a holding potential of −40 mV.

Kv4.2 current, The pipette solution contained (mM) 140 KF, 5 ATP-2Na, 1 EGTA, and 10 HEPEs (pH = 7.4). The external solution contained (mM) 137 NaCl, 5.9 KCl, 2.2 CaCl_2_, 1.2 MgCl_2_, 14 glucose, and 10 HEPEs (pH = 7.4). The Kv4.2 currents were elicited by a 300-ms depolarizing voltage of 10 mV from a holding potential of −80 mV. The steady-state activation of Kv4.2 was measured with the holding potential −80 mV and applied from −80 mV to 70 mV (10-mV increments). Steady-state inactivation of Kv4.2 channel measured with a series of a 300-ms depolarizing test potential of 10 mV followed a 1000-ms prepulse at potentials ranging from −120 to +70 mV with a 5-mV increment.

Data were analyzed using the clampfit (Axon) and Origin 9 software programs. All data points are shown as mean SEM. The significance was estimated using unpaired two-tailed *t*-tests, and a *P* < 0.05 was considered statistically significant. n stands for the number of the separate experimental cells. The IC_50_ value was calculated with Hill logistic equation: *y* = 1 − {(1 − f_max_)/[1 + [(Tx)/IC_50_]_n_}. The steady-state activation relationship was measured using Boltzmann function (*f*(V) = 1/{1 + exp[−(V−V_1/2_)/*k*]}^4^). Steady-state inactivation was measured using the Boltzmann function *f*(V) = 1/{1 + exp[−(V − V_1/2_)/*k*]}.

## Results

### JZTX-V Inhibits I_to_ and Extends APD in Cardiomyocytes

**FIGURE 1:**
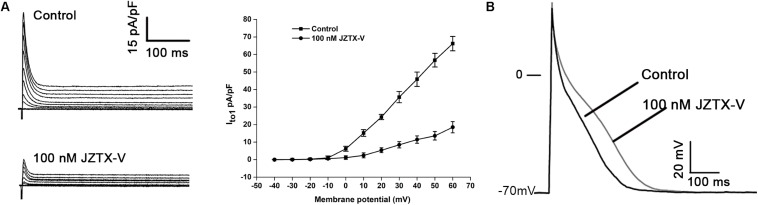
Jingzhaotoxin-V inhibits I_to_ and extended APD in NRVMs isolated from SD rats. **(A)** Representative traces of I_to_ in NRVMs treated with 100 nM JZTX-V. The cell capacitance for the NRVM was 18 ± 3.1 pF. Average I–V relation curves of I_to_ in NRVMs normalized to cell capacitance treated with 100 nM JZTX-V. **(B)** Representative traces of action potential recorded in NRVMs treated with 100 nM JZTX-V. For the AP of NRVMs Treatment with the JZTX-V prolonged APD_1__0_ from 12 ± 1 ms to 15 ± 1.7 ms, APD_5__0_ from 88 ± 6 ms to 109 ± 8 ms, APD_90_ from 245 ± 7 ms to 309 ± 10 ms.

Jingzhaotoxin-V, an inhibitor cystine knot (ICK)-gating modifier, blocks A-type potassium channels in DRG cells with an IC_50_ value of 52.3 nM (Zeng et al., 2008). Based on this finding, we speculate that JZTX-V could affect cardiac AP by inhibiting the I_to_ current. The cell capacitance for the NRVM was 18 ± 3.1 pF. Average I–V relation curves of I_to_ in NRVMs normalized to cell capacitance treated with 100 nM JZTX-V. As shown in Figure 1A, JZTX-V at a concentration of 100 nM inhibited the I_to1_ current of NRVMs by 78 ± 4.32% at 30 mV (*N* = 4). For the AP of NRVMs (at 1 Hz), resting membrane potential was −70 ± 2.3 mV and the amplitude was 105 ± 12 mV in control group and the resting membrane potential in control group was −70 ± 2.3 mV, and the amplitude was 109 ± 12.3 mV. Although JZTX-V treatment modifies the amplitude of the AP, the difference was no statistically significant. JZTX-V (100 nM) prolonged the APD_10_ (123.53 ± 4.23%; APD_50_, 124.34 ± 3.17%; and APD_90_, 126.26 ± 3.23%), compared with that in the vehicle-treated group, but did not affect APD_10__–__50_, and APD_50__–__90_ to a significant extent (*N* = 5; Figure 1B).

### Effect of JZTX-V on the Kv4.2 Current

**FIGURE 2:**
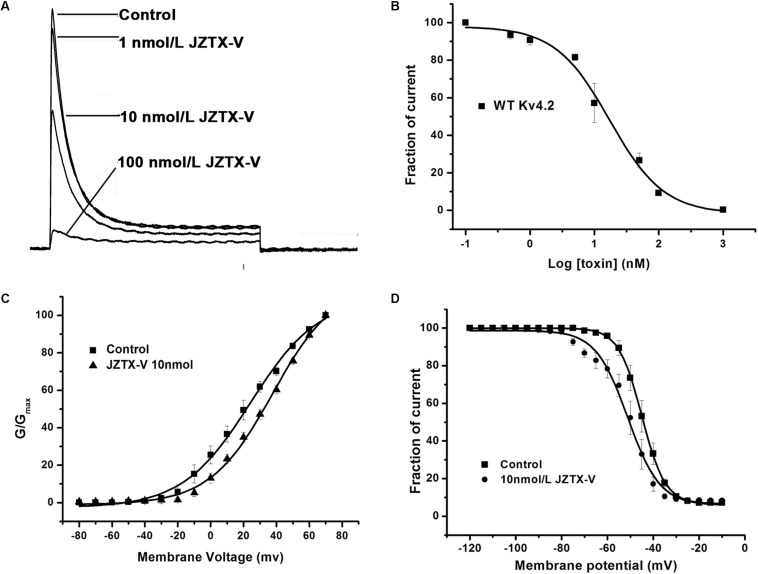
Effects of JZTX-V on WT Kv4.2-kchip2 expressed in HEK293T cells. **(A)** Kv4.2 current traces. Data are normalized to the maximum peak current amplitude. **(B)** The concentration-dependent inhibition of WT Kv4.2 channel by JZTX-V. Data points (mean±SE) were fit with the Hill equation as described under the Section “Materials and Methods.” **(C,D)** Effects of the JZTX-V on normalized steady-state activation and inactivation of WT Kv4.2.

Kv4.2 is a cloned channel believed to underlie the I_to_ current in NRVMs. Here, the I_to_ current was recorded in HEK293t cells transfected with vectors co-expressing the Kv4.2 and KChIP2 subunits. As shown in Figure 2A, 10 nM JZTX-V induced a significant reduction in peak amplitude of the control potassium current by a maximum of 42.8 ± 8.2% (*N* = 7) and 100 nM JZTX-V inhibited the potassium current over 90% (*N* = 7). These inhibitory effects were concentration-dependent, with a half-maximal inhibitory concentration (IC_50_) value of 13 ± 1.7 nM (Figure 2B; *N* = 7). Moreover, addition of JZTX-V (10 nM) led to alterations in the G–V curve of the Kv4.2 potassium channel. V_1/2_ values were +20 mV and +30 mV in the absence and presence of JZTX-V, respectively (Figure 2C). During the steady-state inactivation process, 10 nM JZTX-V induced a slight shift in the Kv4.2 current with a half-maximal inactivation potential (V_in1/2_) shift in the hyperpolarizing direction by 4.5 mV (from −44.5 to −50.0 mV) (Figure 2D).

### Inhibitory Activity of JZTX-V on Kv4.2 Mutants

**FIGURE 3:**
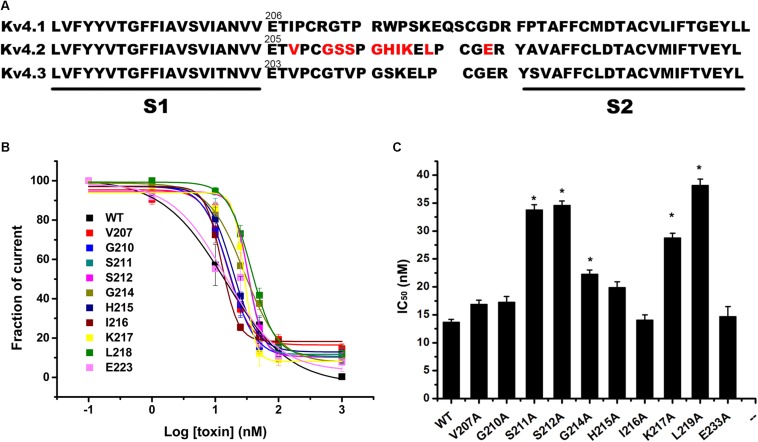
Inhibition effects of site mutants in the S1–S2 linker of Kv4.2 by JZTX-V. **(A)** Sequence alignment of S1–S2 regions of Kv4 subfamily a-subunits (Red were used in alanine-scanning mutagenesis). **(B,C)** IC_50_ values on Kv4.2 channel of JZTX-V and analogs. Data shown are the means ±SEM (*N* = 5). *P < 0.05 compared with WT group.

**FIGURE 4:**
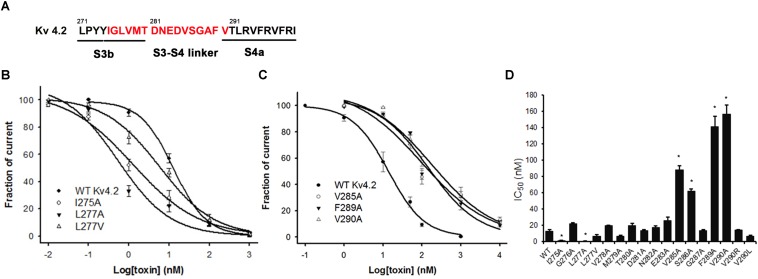
Inhibition effects of site mutants in the S3b and S3–S4 linker of Kv4.2 by JZTX-V. **(A)** S3b–S4 regions of Kv4.2. Red amino acids are those used in alanine-scanning mutagenesis site mutants on the S3b and S3–S4 linker. **(B)** Concentration-response curves for JZTX-V acted on WT-Kv4.2 and the mutants I275A, L277A, and L277V significantly increase affinity to JZTX-V. **(C)** Hydrophobic residues mutants V285A, F289A, and V290A lose affinity to JZTX-V. **(D)** IC_50_ values on S3–S4 linker of Kv4.2 channel mutants of JZTX-V. *P < 0.05 compared with WT group.

The majority of peptide toxins are reported to interact with linkers of transmembrane helices S3 and S4 in the Kv4 channel as gating modifiers (Ebbinghaus et al., 2004; DeSimone et al., 2009; Yuan et al., 2012; Xie et al., 2014). JZTX-V showed strong affinity for the Kv4 channel but low gating modification function. To elucidate the mechanism underlying JZTX-V binding to Kv4.2, the amino acid residues in the S1–S2 linker (Figure 3A) and S3b–S4 linker (Figure 4A) of Kv4.2 were mutated to Ala using the alanine scanning technique. Sequence comparisons revealed more positively charged residues (Arg) on the S1–S2 linker of Kv4.1 relative to Kv4.2, and Kv4.3 (Figure 3A). As shown in Figures 3B,C, all mutants displayed only a slight decrease in JZTX-V binding affinity, suggesting that the S1–S2 linker of Kv4.2 does not play a key role in JZTX-V-mediated inhibition. Alanine scanning on the S3b–S4 led to the identification of five component residues (Ile275, Leu277, Val285, Phe289, and Val290) critical for JZTX-V binding (Figures 4B,C). Mutation of Val285, Phe289, and Val290 reduced sensitivity to the toxin by ∼7-, 11-, and 12-fold with IC_50_ values of 88.6 ± 3.2, 141.2 ± 7.1, and 158.2 ± 5.9 nM, respectively (*N* = 6). Interestingly, two Kv4.2 mutants, Ile275A and Leu277A, displayed markedly increased affinity for JZTX-V (∼10- and 20-fold, respectively). Because Val has similar hydrophobic properties but a shorter β-carbon chain than Leu, we additionally mutated Leu277 to Val. Kv4.2 inhibited L277V mutation with an IC_50_ value of 6.3 nM. Based on the collective findings, we proposed that JZTX-V docked at the S3b–S4 linker of Kv4.2 and the hydrophobic amino acids within the S3b–S4 segment were potentially critical for toxin-mediated inhibition. In contrast to HaTx (Swartz and MacKinnon, 1997) and other well characterized ICK toxins, JZTX-V binding did not appear to require a charged amino acid.

### Effects of JZTX-V Mutants on Kv4.2 Binding

**FIGURE 5:**
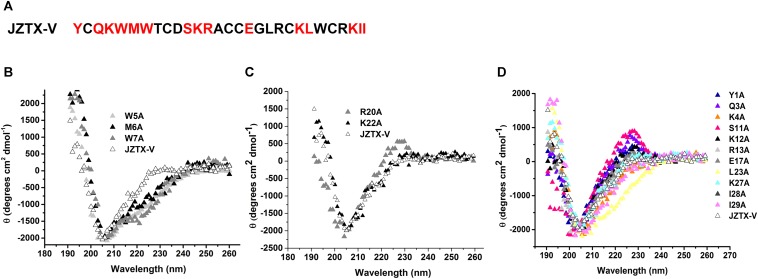
Sequence and CD spectra of JZTX-V. **(A)** Amino acid sequence of JZTX-V and its mutant residues are shaded. **(B–D)** Comparison of the CD spectrum of JZTX-V and mutants with the critical hydrophobic residues **(B)**, and charged residues **(C)**, and other mutants **(D)**.

Jingzhaotoxin-V contains a large hydrophobic patch comprising three aromatic and two aliphatic residues, which may be responsible for binding to the Kv4.2 S3–S4 linker. Fifteen JZTX-V mutants located on the surface of the three-dimensional structure were efficiently synthesized via alanine screening mutagenesis. The structural integrity of JZTX-V and its mutations was determined by measuring the CD spectrum from 260 to 190 nm in 0.01 M sodium phosphate solution (pH 7.0) at room temperature. CD spectra of the JZTX-V mutants almost overlapped completely with that of wild-type toxin, indicating that the mutations do not induce significant alterations in the secondary structure (Figure 5).

**FIGURE 6:**
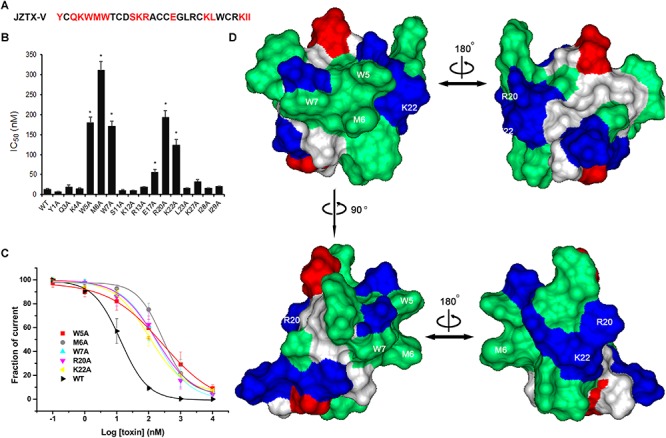
Effects of JZTX-V and analogs on Kv4.2 current. Inhibitory activity of JZTX-V and mutants on Kv4.2 channel. **(A)** the residues mutated to Ala are shaded in red. **(B)** IC_50_ values on Kv4.2 channel of JZTX-V and analogs. **(C)** concentration dependence for the inhibition of five JZTX-V mutants (W5A, M6A, W7A, R20A, and K22A) on Kv4.2. **(D)** bioactive surface profile of JZTX-V binding to Kv4.2. The left and right structures were rotated 180° relative to one another about a vertical axis. *P < 0.05 compared with WT group.

Next, we measured the binding affinities of toxin mutants for the WT Kv4.2 channel expressed in HEK293 cells (Figure 6A). The concentration-dependent curves and estimated IC_50_ values are shown in Figures 6B,C. Alanine replacement of three hydrophobic residues (Trp5, Met6, and Trp7) reduced binding affinity of the toxin by 14-, 24-, and 13-fold, respectively. Surprisingly, mutation of two positively charged residues (R20A and K22A) reduced the toxin binding affinity for Kv4.2 by 15- and 9-fold, respectively. However, the IC_50_ values of other mutants were similar to that of wild-type JZTX-V, suggesting no major involvement of these residues in JZTX-V interactions with Kv4.2 (*N* = 6). These results collectively indicated that positively charged residues (R20 and K22) and an exposed hydrophobic patch formed by W5, M6, and W7 of JZTX-V comprised a functional surface for binding the Kv4.2 channel (Figure 6D).

## Discussion

The Kv4 channels are predominantly located in the heart and central nervous system. In ventricular myocytes of the majority of mammals, Kv4 channels are responsible for the fast recovering transient outward K^+^ current (I_to_) (Tamargo et al., 2004), which plays pivotal roles in the amplitudes and duration of cardiac AP. Inhibitors of Kv4 channels act as anti-arrhythmic drugs via an ionic mechanism (Feng et al., 1997; Ma et al., 2015). In this study, we examined the effects of JZTX-V on the I_to_ current and AP of cardiomyocytes and the potential underlying mechanisms.

Animal toxins target ion channels, and are thus valuable resources with potential therapeutic applications. The Kv4 subfamily from scorpion (Maffie et al., 2013; Xie et al., 2014) and spider venom (Escoubas et al., 2002; Ebbinghaus et al., 2004; Yuan et al., 2007, 2012; DeSimone et al., 2009) has a rich toxin pharmacology. HaTx, originally isolated from venom of the Chilean rose tarantula (*Grammostola spatulata*), inhibits Kv4.2 channels effectively, with similar affinity for Kv2.1 (Swartz and MacKinnon, 1995; Swartz, 2007). Other Kv4 channel inhibitors, Stromatoxin (ScTx1) from *Stromatopelma calceata* and heteroscodratoxins (HmTx1,2) from *Heteroscodra maculata*, are also reported to target Kv2 channels (Escoubas et al., 2002). The Kv4 channel gating-modifying toxin, Ctri9577, is also able to inhibit Kv1.3 potassium channels (Xie et al., 2014). Moreover, heteropodatoxins (HpTx1-3) from *Heteropoda venatoria* (Sanguinetti et al., 1997; Zarayskiy et al., 2005; Desimone et al., 2011), phrixotoxins (PaTx1,2) from *Phrixotrichus auratus* (Diochot et al., 1999), and TLTx1-3 from *Theraphosa blondi* venom (Ebbinghaus et al., 2004) primarily target Kv4 channels, but with IC_50_ values of >100 nM. Therefore, toxins that can effectively and specifically inhibit Kv4.2 are yet to be identified. Previous studies by our group demonstrated that JZTX-V selectively inhibits the Kv4.2 current and displays markedly lower affinity for other potassium channels (Zeng et al., 2007, 2008). In the present investigation, JZTX-V was determined as an efficient inhibitor of Kv4.2 channels (IC_50_ and 13 nM). Under the same set of calculation parameters, ScTx1 is the most potent inhibitor known for Kv4.2 channels with an IC_50_ value of 1.2 nM. However, the selectivity of ScTx1 is far from absolute as it also inhibits Kv2.1 channel with strong affinity (IC_50_ and 12.7 nM; Supplementary Table S1). Furthermore, we have shown for the first time that JZTX-V effectively inhibited the I_to_ current and extended the APD of NRVM. Our results indicated that JZTX-V acted as an efficient and highly selective inhibitor of the Kv4.2 channel, supporting its utility in the treatment of cardiac disorders.

Voltage sensor trapping is the mechanism underlying the modification effects of animal toxins on voltage-dependent gating potassium channels (Wang et al., 2004; Catterall et al., 2007; Swartz, 2007). Several channel mutagenesis studies have shown that toxin binding sites are often complex and involve either a combination of charged and hydrophobic amino acids or charged interactions alone (Swartz, 2007; Zhang et al., 2007; Bosmans et al., 2008). However, only hydrophobic contacts detected in the S3b–S4 linker of Kv4 have been shown to be crucial for channel interactions (DeSimone et al., 2009; Xie et al., 2014). JZTX-V is part of a growing list of peptide-gating modifier toxins that interact with the outer portion of S3b or the S3–S4 linker of voltage-gated ion channels. As shown in Figure 4, the hydrophobic patch formed by V285, F289, and V290 within the S3–S4 linker is critical for interactions with JZTX-V. However, although the toxin binding motifs are structurally conserved, divergence with regard to the composition and arrangement of crucial residues in the voltage sensor were observed (Supplementary Figure S2A). For example, L274 and V275 in the S3b region of Kv4.3 are crucial determinants for binding to toxins PaTx2 and Ctr9577 (Wang et al., 2007; DeSimone et al., 2009). V288 on Kv4.3 promotes binding to Ctri9577 but not PaTx1. Unexpectedly, hydrophobic I275 and L277 within S3b of the Kv4 channel, seem to be a common binding site for PaTx2 and Ctr9577, observed the opposite role for interactions with JZTX-V. Interestingly, alanine mutants (I275A and L277A) of Kv4.2 increased the affinity for JZTX-V by 10- and 20-fold, respectively. N280 and E281 play similar roles in interactions between toxin and the Kv4.3 channel, and mutation of these residues to Ala promoted binding of HpTx2 and Ctri9577 toxins to Kv4.3. However, the reason for the observed increase in binding affinity is unclear. One possibility is limitations for the introduction of secondary conformational and physiochemical changes (DeSimone et al., 2009). Interestingly, Ctri9577 exerts similar potency as PaTx1, although alanine mutants (N280A) of the Kv4.3 channel display decreased Ctri9577 affinity and the E281A mutant shows higher Ctri9577 sensitivity. The results indicated that diverse toxins use partially overlapping bioactive surfaces to target the S3b–S4 linker of the Kv4 channel. On the one hand, hydrophobic interactions are common and improve toxin binding to Kv4, and on the other hand, the steric effects of several residues on S3–S4 linker disrupt the binding of toxins owing to diverse three-dimensional structures.

Toxins bound to Kv4 channels show a highly conserved three-dimensional structure. One of the striking structural features of voltage sensor toxins is their amphipathic nature (Supplementary Figure S2B), with one face containing a cluster of hydrophobic residues (green) that exhibit an unusually high degree of solvent exposure surrounded by basic (blue), acidic (red), and other highly polar residues. Although the overall fold and amphipathic nature of the voltage sensor toxins are similar, a number of differences exist, in particular for residues comprising the hydrophobic surface. As shown in Supplementary Figure S2B, four blockers of Kv4 channels, HpTx (1,2), ScTx1, and TLTx1, show low sequence similarity with JZTX-V but contain a common hydrophobic surface. In HaTx1, the hydrophobic surface comprises Y4, L5, F6, Y27, and W30, with Y4, F6, and W30 forming an aromatic sandwich around L5 (Supplementary Figure S2B). Four hydrophobic residues, W30, M5, F6, and Y7, forming the hydrophobic surface of ScTx1 are located in a straight line. The structure of HpTx1 is similar to that of HaTx1, with L6, F7, W25, and L28 occupying positions equivalent to L5, F6, Y27, and W30, respectively. HpTx2 lacks the equivalent of Y4 in HaTx1 but contains three additional hydrophobic residues (W30, Y20, and V21) that contribute to the solvent-exposed hydrophobic surface. As shown in Supplementary Figure S1, PaTx1 and PaTx2 show high sequence similarity to JZTX-V and share the same hydrophobic surface encompassing W5, M6, W7, and W24 (Supplementary Figure S2B). Alanine scanning mutagenesis of JZTX-V has provided an essentially complete map of the functionally important surfaces of the toxin. The active face of the toxin contains the solvent-exposed hydrophobic surface, which is involved in interactions with the Kv4.2 channel. These findings validated the theory that hydrophobic force plays an important role in toxin binding to Kv4 channels. The active hydrophobic surface of SGTx provides residues that interact with the Kv channel (Wang et al., 2004). Ctri9577, a novel toxin from scorpion *Chaerilus tricostatus* containing few hydrophobic residues, shows lower affinity for Kv4.2 than other toxins, with an IC_50_ value of 1.34 μM (Supplementary Table S1). Although studies on channel toxins have highlighted the importance of protein-protein interactions in toxin binding to voltage-activated channels, it is also possible that lipids constitute an important part of the toxin receptor (Lee et al., 2003; Schmidt and MacKinnon, 2008; Swartz, 2008). JZTX-V contains four basic residues (K4, R20, K22, and R26) positioned on the side of hydrophobic patch and two basic residues (R13 and K26) on the opposite surface. Phospholipid membrane binding experiments reveal that basic residues in the functionally important surface of JZTX-V interact with negatively charged phospholipids (Zeng et al., 2007). Alanine mutants of basic residues (R20 and K22) decreased the toxin binding affinity by 15- and 9-fold, respectively. We speculate that electrostatic interactions mediated by R20 and K22 of JZTX-V are involved in binding to phospholipid membranes. In contrast to the results obtained for other spider toxins, mutation of acid residues failed to alter the affinity of toxin for Kv4.2 (Cohen et al., 2005; Rong et al., 2011).

## Conclusion

In conclusion, JZTX-V effectively inhibited the I_to_ current and extended theAPD. The molecular mechanism underlying the inhibitory action of JZTX-V on I_to_ may involve recognition of a conserved binding motif on the S3–S4 linker. Hydrophobic force plays an important role in interactions between the toxin and channel, and the extracellular conformational space of Kv4 modulates toxin affinity. Moreover, the phospholipid membrane may be involved in binding to toxin. These findings broadened our understanding of the effects of JZTX-V on cardiac electrophysiology and further clarified the molecular mechanisms underlying the inhibitory action of JZTX-V on I_to_ potassium channels, supporting its development as a potential novel antiarrhythmic drug.

## Ethics Statement

SD rats (Hunan SJA Laboratory Animal Co., Ltd., Changsha, China) were used according to the guidelines of the National Institutes of Health for Care and Use of Laboratory Animals. The experiments were approved by the Animal Care and Use Committee of Hunan Normal University.

## Author Contributions

YZ and JL designed the research. YZ and JH performed the research and contributed new reagents or analytic tools. YZ and MR analyzed the data. YZ and XZ wrote the manuscript.

## Conflict of Interest Statement

The authors declare that the research was conducted in the absence of any commercial or financial relationships that could be construed as a potential conflict of interest.

## Abbreviations

HaTx: hanatoxin
ICK motif: inhibitor cystine knot motif
JZTX-V: tarantula toxin jingzhaotoxin-V
KChIP: Kv4 channel-interacting protein
WT: wild type.
