# Supplementary material for: JZTX-V Targets the Voltage Sensor in Kv4.2 to Inhibit Ito Potassium Channels in Cardiomyocytes
Source: Front Pharmacol. 2019 Apr 16;10:357. doi: 10.3389/fphar.2019.00357 (PMC6476928; doi:10.3389/fphar.2019.00357)
Supplement: Supplementary file 1 [file Data_Sheet_1.docx]

Supplementary Material

JZTX-V Targets the Voltage Sensor in Kv4.2 to Inhibit Ito Potassium Channels in Cardiomyocytes

**Yiya Zhang^1,2,3^*, Ji Luo^3^, Juan He^3^, Mingqiang Rong^3^，Xiongzhi Zeng^3^***

*** Correspondence:** Yiya Zhang, yiya0108@csu.edu.cn; Xiongzhi Zeng, [zengxz@hunnu.edu.cn](mailto:zengxz@hunnu.edu.cn)


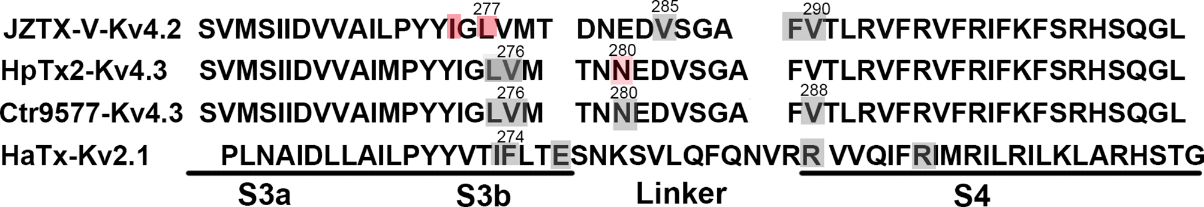


**Supplementary Figure 1.** Sequence alignment of S3b–S4 regions of two mammalian voltage-gated K^+^ channel Kv4.2, Kv4.3 and Kv2.1. The crucial residues, whose mutants increase the toxin affinity, are shaded in red. The crucial residues, whose mutants decrease the toxin affinity, are shaded in gray.


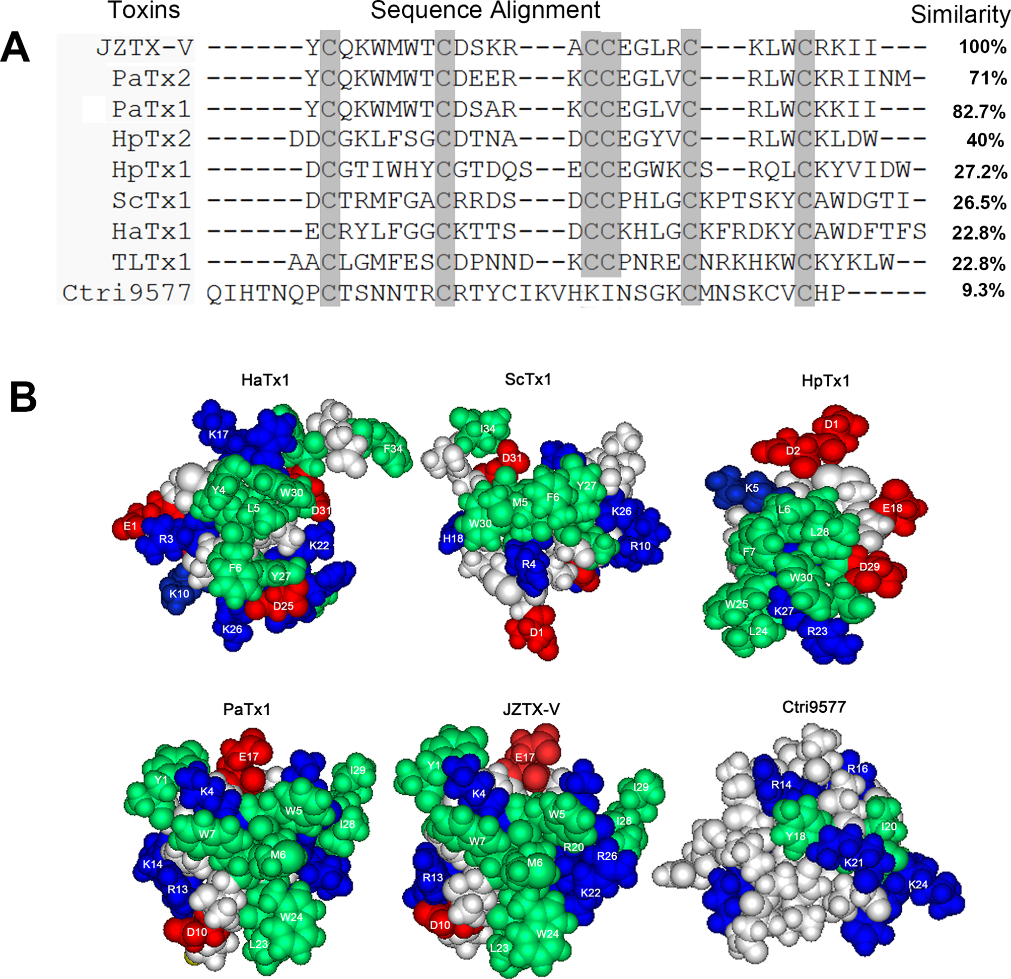


**Supplementary Figure 2,** Sequence alignment and Three-dimensional structure of Kv channel inhibitors. A, Sequence alignment of nine voltage-gated K^+^ channel inhibitors. **B,** Three-dimensional structure of HaTx1 (1D1H), ScTx1, HpTx1 (1emx), PaTx1 (1V7F), JZTX-V and Ctri8557. Model of ScTx1 were obtained by homology modeling based on the structure of JZTX-XI determined by NMR (PDB code 2a2v). Model of JZTX-V were obtained by homology modeling based on the structure of PaTx1 determined by NMR (PDB code 1V7F). Model of Ctri8557 were obtained by homology modeling based on the structure of Ia2 determined by NMR (PDB code 1lir). Residues are colored according to their properties. Blue, basic (Arg, Lys); Red, acidic (Glu, Asp); Green, hydrophobic (Ala, Ile, Leu, Met, Phe, Pro, Trp, Tyr, and Val).

**Supplementary Table 1.**Activity of spider toxins on Kv2 and Kv4 channel subtypes Activities of spider toxins against different subtypes of Kv2 and Kv4 potassium channels are reported as IC_50_ (nanomolar) or as percentage of current inhibition at a single dose (nanomolar). Affinity (IC_50_) measurements obtained in electrophysiological experiments were obtained using a single site relation model (Hill relation, n_H_ = 1). ‘_O_’ is the channels expressed in *X. laevis oocytes*; ‘_H_’ is the channels expressed in HEK293 cells.

| toxin | kv2.1 | kv4.1 | kv4.2 | kv4.3 | note |
| --- | --- | --- | --- | --- | --- |
| Ctri9577 |  | 14% (1µM）_H_ | 10% (1µM）_H_ | 43% (1µM）_H_ | [*Chaerilus tricostatus*](http://www.ncbi.nlm.nih.gov/Taxonomy/Browser/wwwtax.cgi?id=1055734) [*^34^*](#_ENREF_34) |
| ScTx1 | 12.7 nM _O_ | No _O_ | 1.2 nM _O_ |  | *Stromatopelma calceatum* [*^31^*](#_ENREF_31) |
| HmTx1 | 23% (100nM) _O_ | 280 nM _O_ | 39% (300nM) _O_ | 43%(300nM) _O_ | [*Heteroscodra maculata*](http://www.ncbi.nlm.nih.gov/Taxonomy/Browser/wwwtax.cgi?id=268413) [*^31^*](#_ENREF_31) |
| HaTx1 | 42 nM _O_ |  | 70% (500nM) _O_ |  | [*Grammostola rosea*](http://www.ncbi.nlm.nih.gov/Taxonomy/Browser/wwwtax.cgi?id=432528) [*^33^*](#_ENREF_33) |
| PaTx1 | 6% (500nM) | 39% (250nM) | 5 nM _O_ | 28 nM _O_ | *Paraphysa scrofa* [*^37^*](#_ENREF_37) |
| PaTx2 | 8% (500nM) | 20% (250nM) | 34 nM _O_ | 71 nM _O_ | *Paraphysa scrofa* [*^37^*](#_ENREF_37) |
| HpTx1 |  |  | 100 nM _O_ |  | [*Heteropoda venatoria*](http://www.ncbi.nlm.nih.gov/Taxonomy/Browser/wwwtax.cgi?id=152925) [*^34^*](#_ENREF_34) |
| HpTx2 |  |  | 100 nM _O_ | 2.3µM _O_ | [*Heteropoda venatoria*](http://www.ncbi.nlm.nih.gov/Taxonomy/Browser/wwwtax.cgi?id=152925) [*^34^*](#_ENREF_34) |
| HpTx3 |  |  | 67 nM _O_ |  | [*Heteropoda venatoria*](http://www.ncbi.nlm.nih.gov/Taxonomy/Browser/wwwtax.cgi?id=152925) [*^34^*](#_ENREF_34) |
| JZTX-XIII 0.47µM _H_ | | 1.17µM _H_ | 53% (5µM) _H_ |  | *Chilobrachys guangxiensis* [*^27^*](#_ENREF_27) |
| TLTx1 |  |  | 139 nM _H_ |  | [*Theraphosa blondi*](http://www.ncbi.nlm.nih.gov/Taxonomy/Browser/wwwtax.cgi?id=260533) [*^47^*](#_ENREF_47) |
| JZTX-V | 35% (5µM) _O_ | 25% (5µM) _O_ | 13 nM _H_ |  | *Chilobrachys guangxiensi**s*[*^18^*](#_ENREF_18) |
